# Author Correction: E3 ligase FBXW7 is critical for RIG-I stabilization during antiviral responses

**DOI:** 10.1038/s41467-026-70926-x

**Published:** 2026-03-30

**Authors:** Yinjing Song, Lihua Lai, Zhenlu Chong, Jia He, Yuanyuan Zhang, Yue Xue, Yiwei Xie, Songchang Chen, Ping Dong, Luoquan Chen, Zhimin Chen, Feng Dai, Xiaopeng Wan, Peng Xiao, Xuetao Cao, Yang Liu, Qingqing Wang

**Affiliations:** 1 Institute of Immunology, Zhejiang University School of Medicine, Hangzhou, China; 2 The Children’s Hospital, Zhejiang University School of Medicine, Hangzhou, China; 3 Department of Neurobiology, Zhejiang University School of Medicine, Hangzhou, China; 4 National Key Laboratory of Medical Molecular Biology and Department of Immunology, Chinese Academy of Medical Sciences, Beijing, China

Correction to: *Nature Communications* 10.1038/ncomms14654, published online 13 March 2017

In the version of the article initially published, there were unintentional error in some figures. In Fig. 1d, e: the white bars should represent the Control siRNA, and the black bars should represent the FBXW7 siRNA, rather than the reverse. The image in Supplementary Fig. 1f depicting CD11c^+^ and CD11b^+^Gr-1^+^ cells for the Lysm^+^FBXW7^f/f^ group was unintentionally duplicated from the FBXW7^f/f^ group. The image in Supplementary Fig. 6b showing DAPI staining for the VSV 3 h group was mistakenly duplicated from the VSV 0 h group. These errors do not affect the conclusions of the study or the validity of the reported findings.

Due to the age of the article, the figures cannot be updated directly. Revised figures are available as Supplementary Information accompanying this amendment. This notice serves to correct the article.

## Supplementary information

Corrected Fig. 1d, e, Supplementary Figs. 1f, g, 6b

