# Supplementary material for: E3 ligase FBXW7 is critical for RIG-I stabilization during antiviral responses
Source: Nat Commun. 2017 Mar 13;8:14654. doi: 10.1038/ncomms14654 (PMC5355826; doi:10.1038/ncomms14654)
Supplement: Supplementary Information — Supplementary Figures and Supplementary Tables [file ncomms14654-s1.pdf]

Supplemental Figure 1

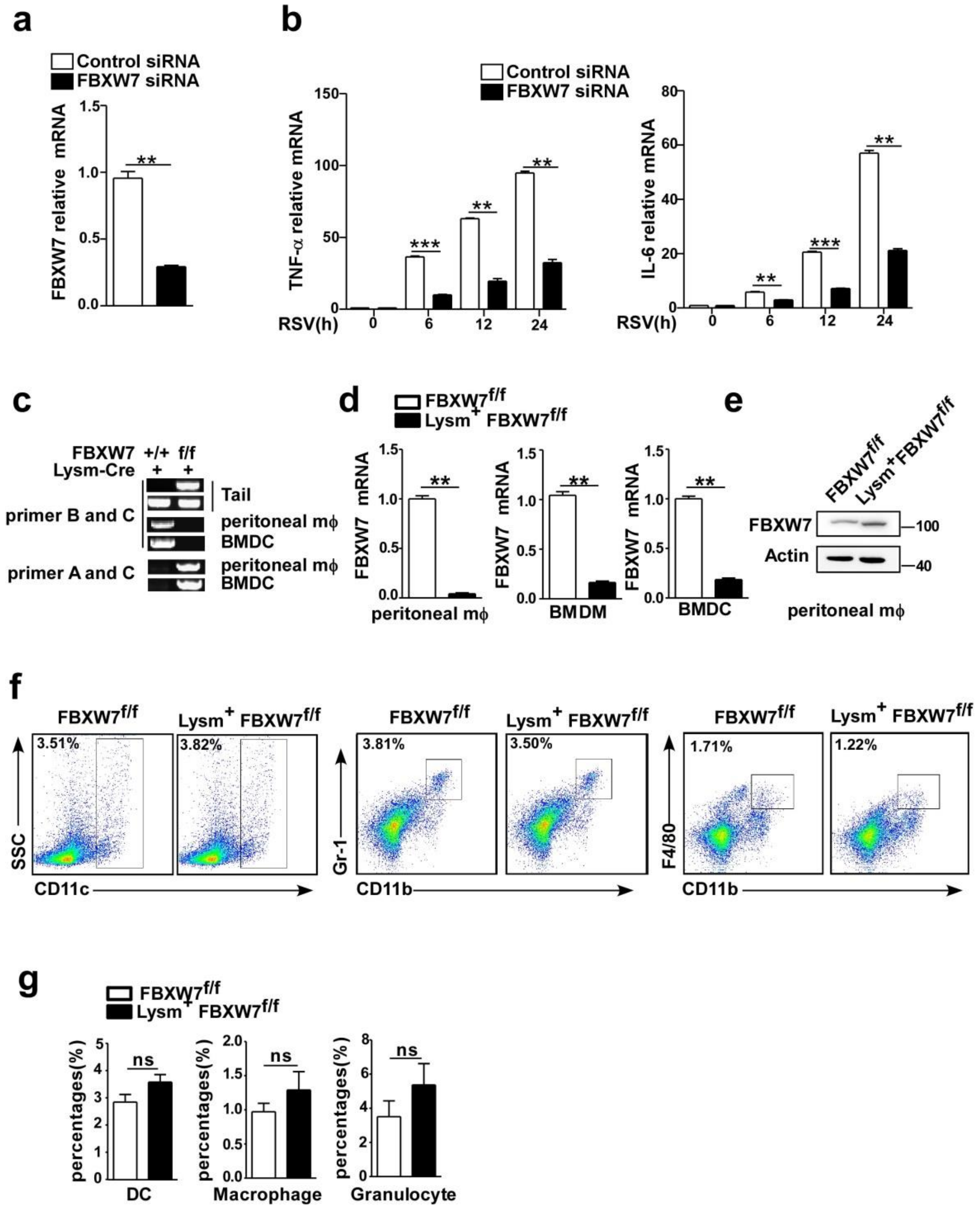

Supplementary Figure 1. FBXW7-deficient mice are more sensitive to RNA virus infection by producing more type I interferon. (a) Q-PCR analysis of FBXW7 expression in THP-1 cells transfected for 36 hr with 60nM FBXW7 siRNA (siFBXW7). **(b)** Q-PCR analysis of TNF-α and IL-6 expression in THP-1 cells transfected for 36 hr with siFBXW7 and infected with RSV. **(c)** PCR analysis of "floxed" and WT alleles (497 and 315 bp, respectively) using primer B and C, and determination of excision of exons 5 and 6 by primers A and C, which amplify 662 bp in the $Lysm^{+}FBXW7^{f/f}$ macrophages and DCs. **(d)** Q-PCR analysis of FBXW7 expression in peritoneal macrophages, BMDM and DCs. **(e)** Western blot analysis of FBXW7 protein in macrophages. **(f)** Flow cytometry analysis of $CD11c^{+}$ DC, $CD11b^{+}Gr\text{-}1^{+}$ granulocytes and $F4/80^{+}CD11b^{+}$ macrophages in spleen from $FBXW7^{f/f}$ and $Lysm^{+}FBXW7^{f/f}$ mice. **(g)** Statistical analysis of percentages of DCs, macrophages and granulocytes in spleen from $FBXW7^{f/f}$ and $Lysm^{+}FBXW7^{f/f}$ mice by flow cytometry (n=6 per group). Data are mean ± s.e.m and are representative of three independent experiments. Student's *t* test was used for statistical calculation. **P<0.01, ***P<0.001.

Supplemental Figure 2

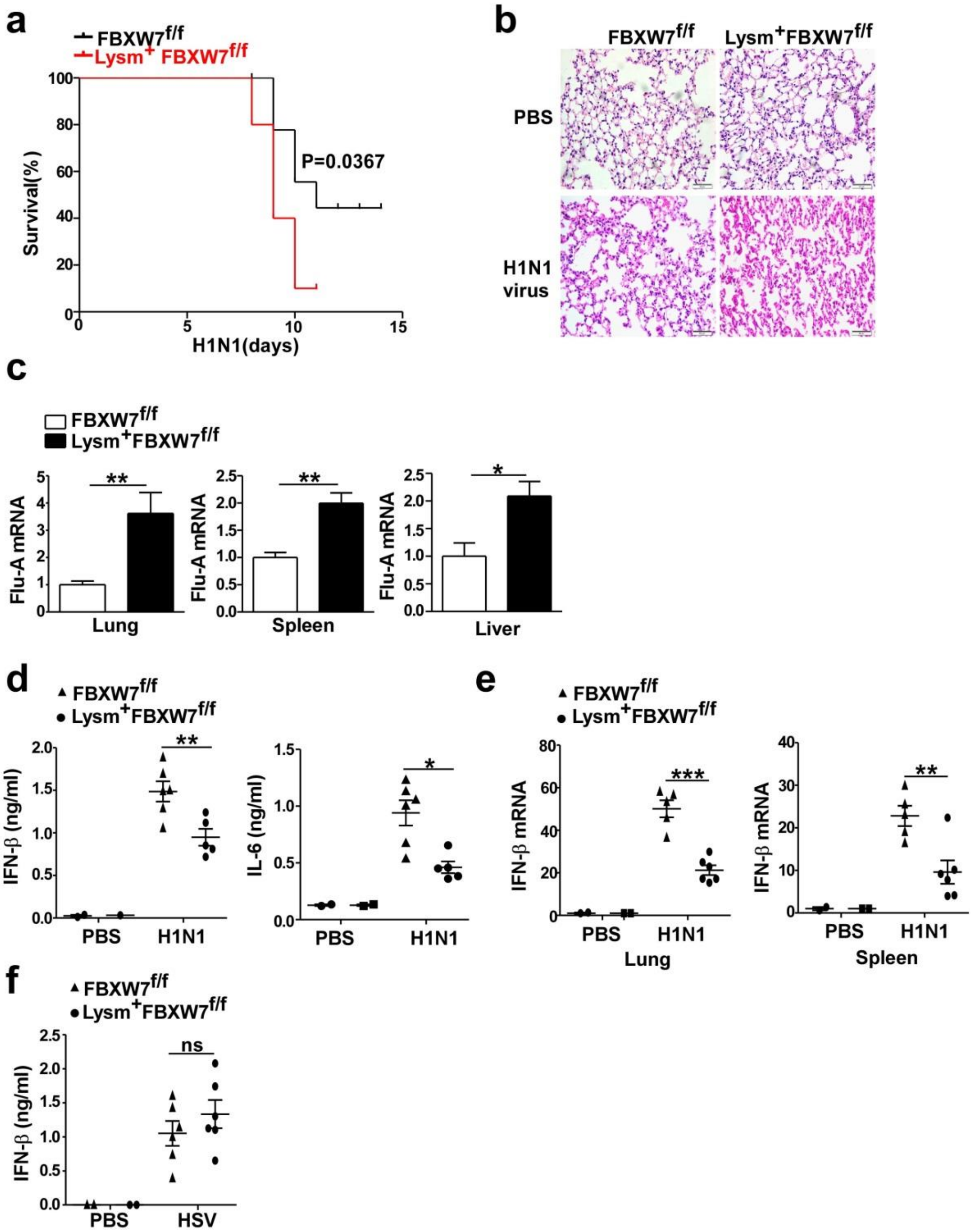

Supplementary Figure 2. FBXW7 inhibits VSV infection and increases type I interferon production *in vivo*. (a) Survival assay of ~7-week-old FBXW7$^{f/f}$ and Lysm^{+}FBXW7$^{f/f}$ mice infected with H1N1 virus (2×10^{3} pfu) by intranasal inoculation. n=9 per group. Kaplan-Meier survival curves were generated and analyzed**. (b)** Lung histology of FBXW7$^{f/f}$ and Lysm^{+}FBXW7$^{f/f}$ mice in response to H1N1 virus. Scale bar, 50 μm. Hematoxylin and eosin staining of lung tissues from mice infected with H1N1 (2×10^{3} pfu) by intranasal inoculation for 6 days. **(c)** Q-PCR analysis of Flu A expression in organs from FBXW7$^{f/f}$ and Lysm^{+}FBXW7$^{f/f}$ mice in **(b)** was shown. **(d)** ELISA assay of cytokine production in sera from FBXW7$^{f/f}$ and Lysm^{+}FBXW7$^{f/f}$ mice in **(b)**. **(e)** Q-PCR analysis of IFN-β expression in spleen and lung from FBXW7$^{f/f}$ and Lysm^{+}FBXW7$^{f/f}$ mice in **(b)**. **(f)** ELISA assay of cytokine production in sera from FBXW7$^{f/f}$ and Lysm^{+}FBXW7$^{f/f}$ mice (n=6 per group) intraperitoneally injected with HSV. Data are mean ± s.e.m and are representative of three independent experiments. Student's *t* test was used for statistical calculation. *P<0.05; **P<0.01; ***P<0.001.

Supplementary Figure 3

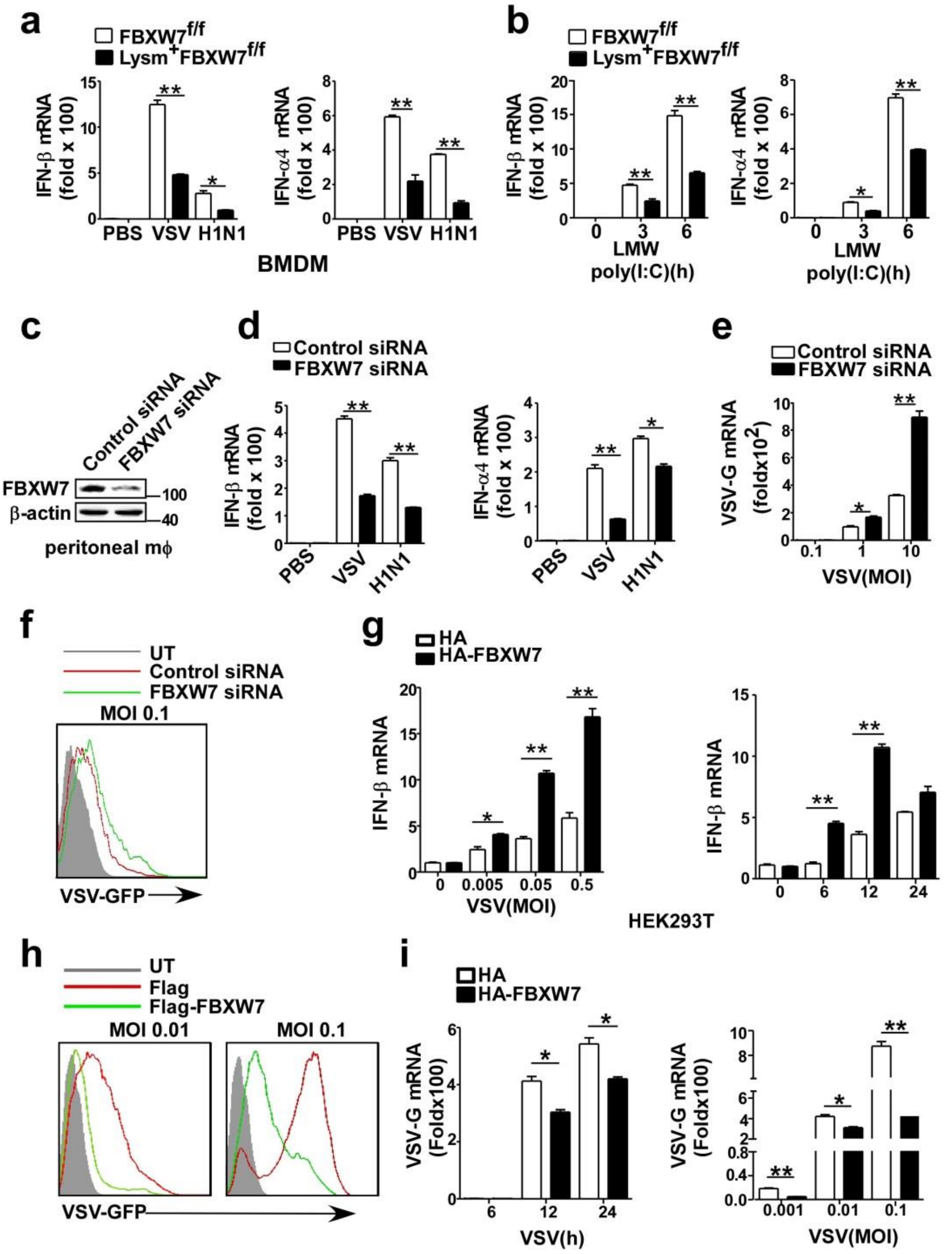

Supplementary Figure 3. FBXW7 inhibits RNA virus infection and promotes type I interferon production *in vitro*. (a) Q-PCR analysis of IFN-β, IFN-α4 mRNA expression in BMDMs from $FBXW7^{f/f}$ and $Lysm^{+}FBXW7^{f/f}$ mice. Cells were infected with VSV or H1N1 virus. **(b)** Q-PCR analysis of IFN-β, IFN-α4 mRNA expression in $FBXW7^{f/f}$ and $Lysm^{+}FBXW7^{f/f}$ peritoneal macrophages transfected with LMW-poly (I:C) by Jetprime for 3, 6 hr. **(c)** Immunoblot analysis of the knockdown of exogenous FBXW7 in peritoneal macrophages. **(d)** Q-PCR analysis of IFN-β, IFN-α4 mRNA expression in FBXW7-silenced peritoneal macrophages infected with VSV or H1N1 virus. **(e)** Q-PCR analysis of VSV-G mRNA expression in FBXW7-silenced macrophages infected with VSV-GFP at a MOI of 0.1,1 or 10. **(f)** Flow cytometry analysis of GFP^{+} cells in FBXW7-silenced macrophages infected with VSV-GFP. **(g)** Q-PCR analysis of IFN-β mRNA in FBXW7-overexpressed HEK293T cells infected with VSV at indicated MOI and time points. **(h)** Flow cytometry analysis of GFP^{+} in HEK293T cells overexpressing FBXW7 infected with VSV-GFP for 12 hr. **(i)** Q-PCR analysis of VSV-G mRNA in FBXW7-overexpressed HEK293T cells infected with VSV at indicated MOI and time points. Data are mean ± s.e.m and are representative of three independent experiments. Student's *t* test was used for statistical calculation. *P<0.05; **P<0.01; ***P<0.001.

Supplementary Figure 4

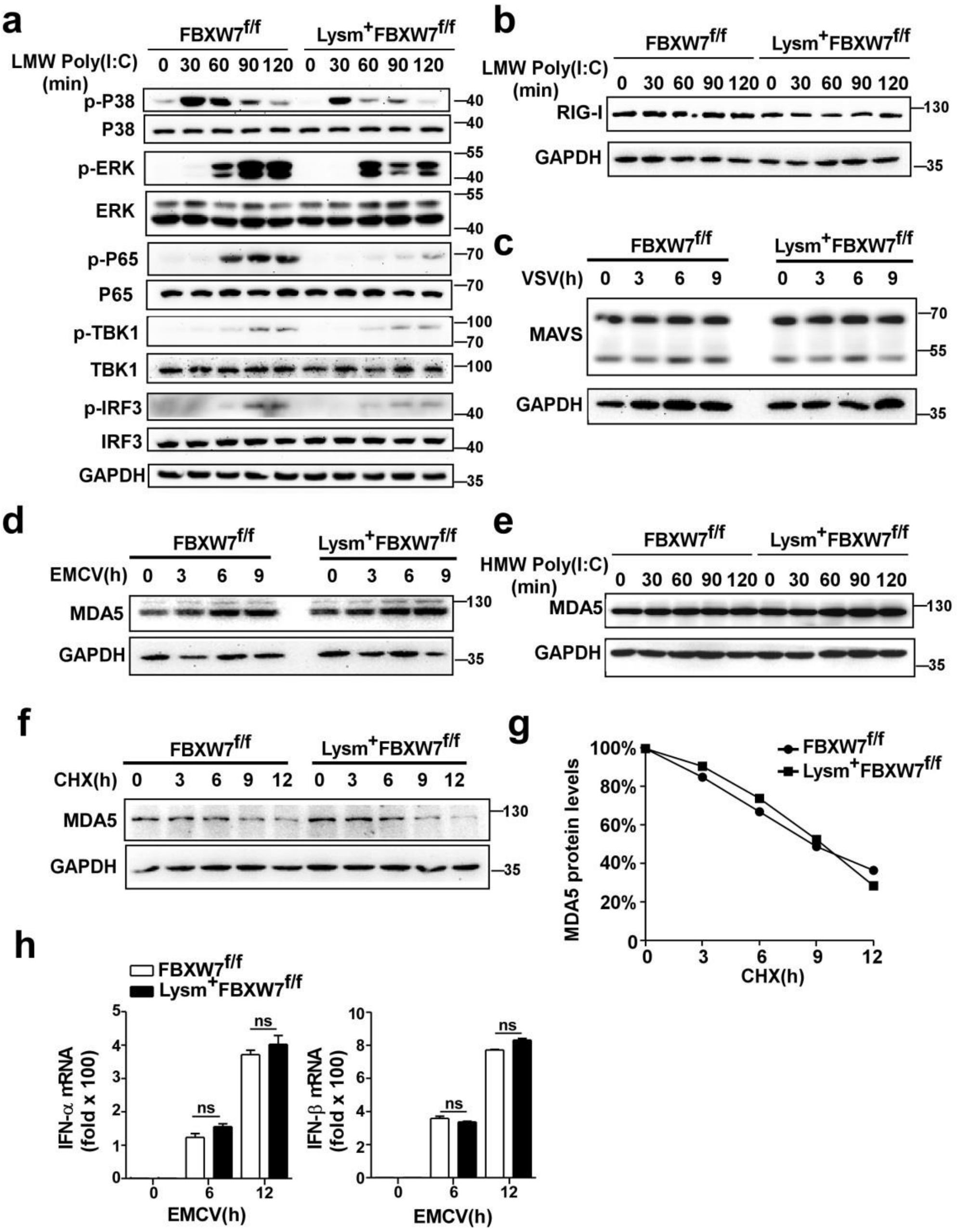

Supplementary Figure 4. FBXW7 positively regulates RIG-I signal pathway through protecting RIG-I from degradation. (a) Immunoblot analysis of phosphorylated or total proteins in lysates of FBXW7$^{f/f}$ and Lysm^{+}FBXW7$^{f/f}$ peritoneal macrophages transfected for indicated hours with LMW-poly (I:C). **(b)** Immunoblot analysis of RIG-I in lysates of FBXW7$^{f/f}$ and Lysm^{+}FBXW7$^{f/f}$ macrophages transfected with LMW-poly (I:C). **(c)** Immunoblot analysis of MAVS in lysates of FBXW7$^{f/f}$ and Lysm^{+}FBXW7$^{f/f}$ macrophages infected for indicated hours with VSV. **(d)** Immunoblot analysis of MDA5 in lysates of FBXW7$^{f/f}$ and Lysm^{+}FBXW7$^{f/f}$ macrophages infected for indicated hours with EMCV. **(e)** Immunoblot analysis of MDA5 in lysates of FBXW7$^{f/f}$ and Lysm^{+}FBXW7$^{f/f}$ macrophages transfected for indicated hours with HMW-poly (I:C). **(f)** Immunoblot analysis of MDA5 in lysates of FBXW7$^{f/f}$ and Lysm^{+}FBXW7$^{f/f}$ peritoneal macrophages treated with CHX (40 μg/ml) for indicated hours after infection with EMCV for 1 hr. **(g)** Quantification of relative MDA5 levels is shown in the right panel. **(h)** Q-PCR analysis of IFN-β, IFN-α4 mRNA expression in FBXW7$^{f/f}$ and Lysm^{+}FBXW7$^{f/f}$ peritoneal macrophages infected with EMCV. Data are mean ± s.e.m and are representative of three independent experiments. Student's *t* test was used for statistical calculation. ns, not significant.

Supplementary Figure 5

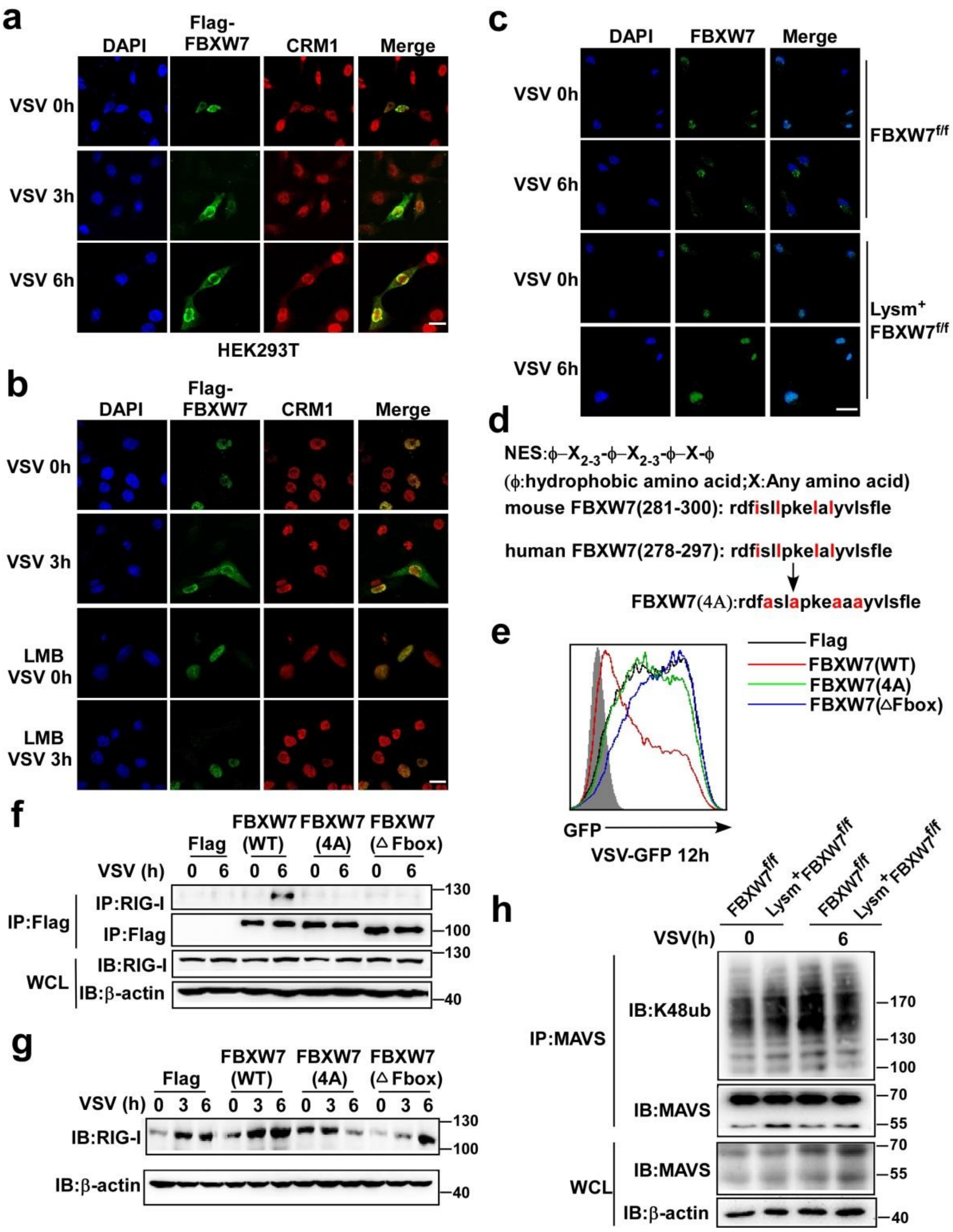

Supplementary Figure 5. FBXW7 translocates into the cytoplasm to interact with RIG-I and exert the antiviral function. (a) Confocal microscopy images of HEK293T cells transfected with Flag-FBXW7 and infected for indicated hours with VSV and labeled with antibodies to the appropriate protein. Scale bar, 20 μm. **(b)** Cofocal microscopy images of HEK293T cells transfected with Flag-FBXW7 and pretreated with 30 μM Leptomycin B (LMB) for 4 hr, then infected for indicated hours with VSV and labeled with antibodies to the appropriate protein. Scale bar, 20 μm. **(c)** Confocal microscopy images of FBXW7$^{f/f}$ and Lysm^{+}FBXW7$^{f/f}$ BMDM infected for indicated hours with VSV. Scale bar, 20 μm. **(d)** Sequence alignment of FBXW7 shows nuclear export signal (NES) (Φ-x$_{2\text{-}3}$-Φ-x$_{2\text{-}3}$-Φ-x-Φ, "Φ" is a hydrophobic residue and "x" is any other amino acid). **(e)** Flow cytometry analysis of GFP^{+} in HEK293T cells overexpressed FBXW7 (wt) or FBXW7 (mutant) and infected with VSV-GFP for 12 hr. **(f)** Coimmunoprecipitation and immunoblot analysis of HEK293T cells transfected for 24 hr with Flag-FBXW7 and infected with VSV. **(g)** Immunoblot analysis of HEK293T cells transfected for 24 hr with Flag-FBXW7 (wt) or Flag-FBXW7 (mutant) and infected with VSV.

Supplementary Figure 6

a

PTPN11 (SHP2)

VSV(h)	0	3
Score	9.85	196.64
Peptides	2	15

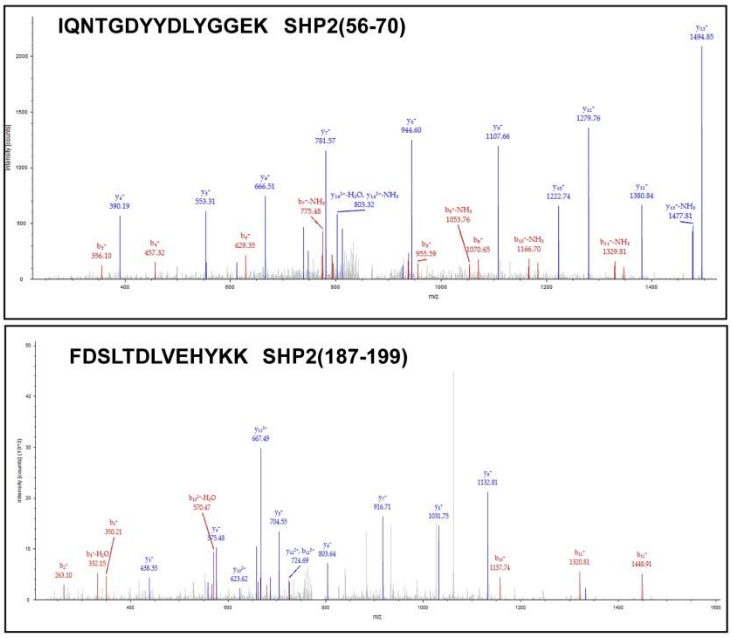

b

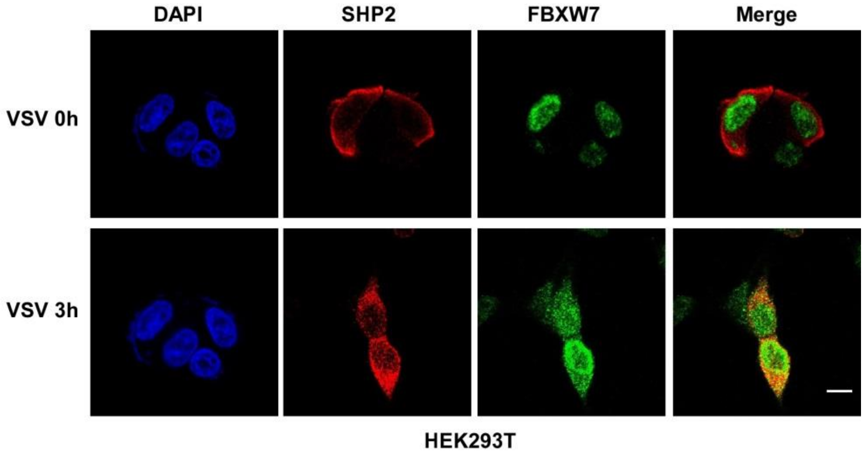

Supplementary Figure 6. FBXW7 interacts with SHP2. (a) The information of SHP2 in FBXW7-interacting proteins identified by mass spectrometry. **(b)** Confocal microscopy imaging of HEK293T cells transfected with Flag-FBXW7 and Myc-SHP2 for 24 hr, then infected for indicated hours with VSV and labeled with antibodies to the appropriate protein. Scale bar, 10 μm.

Supplementary Figure 7

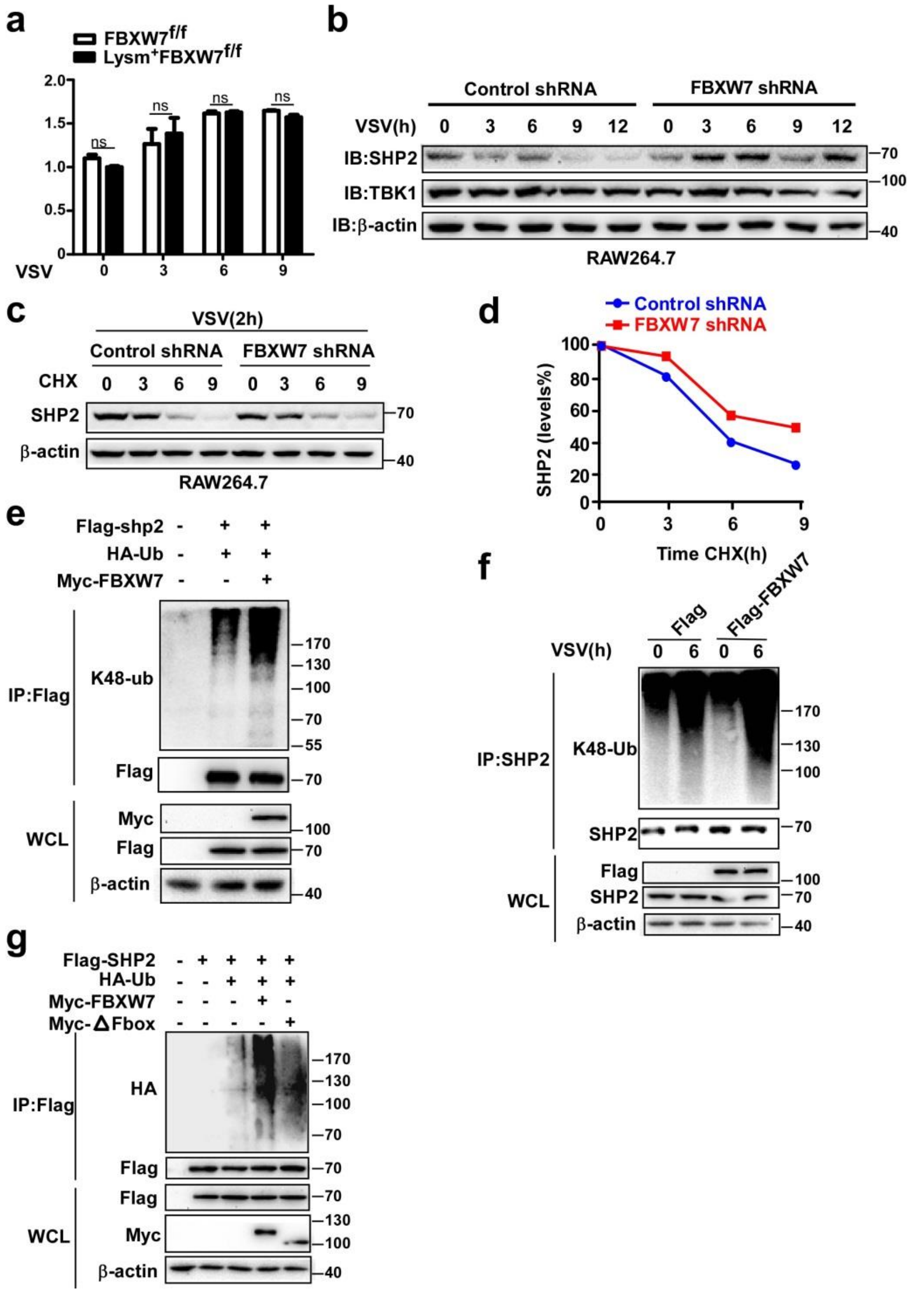

Supplementary Figure 7. FBXW7 mediates the degradation and ubiquitination of SHP2. **(a)** Q-PCR analysis of SHP2 mRNA expression in FBXW7$^{f/f}$ and Lysm^{+}FBXW7$^{f/f}$ peritoneal macrophages infected for indicate hours with VSV. Data are mean ± s.e.m and are representative of three independent experiments. Student's *t* test was used for statistical calculation. ns, not significant. **(b)** Immunoblot analysis of SHP2 in lysates of FBXW7-silenced RAW 264.7 cells infected with VSV. **(c, d)** Immunoblot analysis of SHP2 in lysates of FBXW7-silenced macrophages treated with CHX (40 μg/ml) for indicated hours after infection with VSV for 1 hr. The protein level of SHP2 was shown in the right panel, which was relative to β-actin protein. **(e)** Immunoblot analysis of the K-48 ubiquitination of SHP2 in HEK293T cells cotransfected with Flag-SHP2, HA-ub, along with increasing concentrations (wedge) of vectors for the Myc-FBXW7 constructs and treated with MG132 before cell harvest. **(f)** Immunoblot analysis of the K-48 ubiquitination of SHP2 in HEK293T cells overexpressing FBXW7 and infected with VSV and treated with MG132 before cell harvest. **(g)** Immunoblot analysis of the ubiquitination of SHP2 in HEK293T cells cotransfected with Flag-SHP2, HA-ub, along with Myc-FBXW7 or Myc-ΔFBOX vector and treated with MG132 before cell harvest.

Supplementary Figure 8

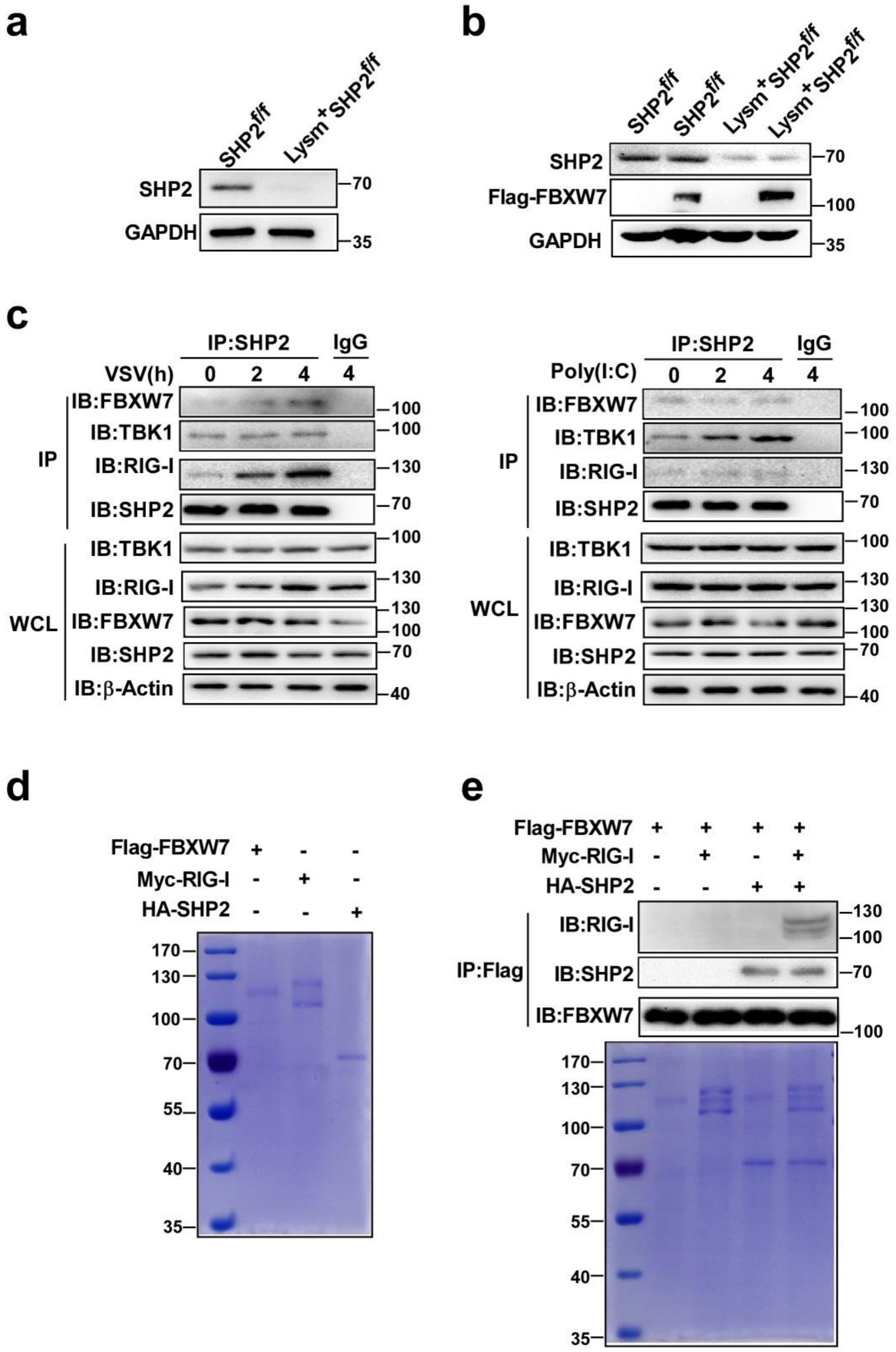

Supplementary Figure 8. Interaction of FBXW7 with RIG-I and SHP2. (a) Immunoblot analysis of SHP2 expression in SHP2$^{f/f}$ and Lysm^{+}SHP2$^{f/f}$ peritoneal macrophages. **(b)** Immunoblot analysis of SHP2 and Flag-FBXW7 expression in SHP2$^{f/f}$ and Lysm^{+}SHP2$^{f/f}$ peritoneal macrophages. **(c)** Immunoblot analysis of RAW264.7 infected with VSV or stimulated with LMW-poly (I:C) for indicate hours, followed by immunoprecipitation with SHP2-conjugated agarose or immunoglobulin G (IgG)-conjugated agarose. **(d)** Coomassie brilliant blue staining of purified Flag-FBXW7, Myc-RIG-I, HA-SHP2 tag-proteins from HEK293T cells transfected with Flag-FBXW7, Myc-RIG-I, HA-SHP2 respectively and infected with VSV for 6 hours. **(e)** The purified fusion protein of Flag-FBXW7 was mixed and incubated with Myc-RIG-I or/and HA-SHP2 protein, followed by immunoprecipitation with Flag-M2 beads. Here shows the result of Immunoblot and Coomassie brilliant blue staining of Flag-FBXW7, Myc-RIG-I, HA-SHP2 tag-protein interaction *in vitro*.

Supplementary Figure 9

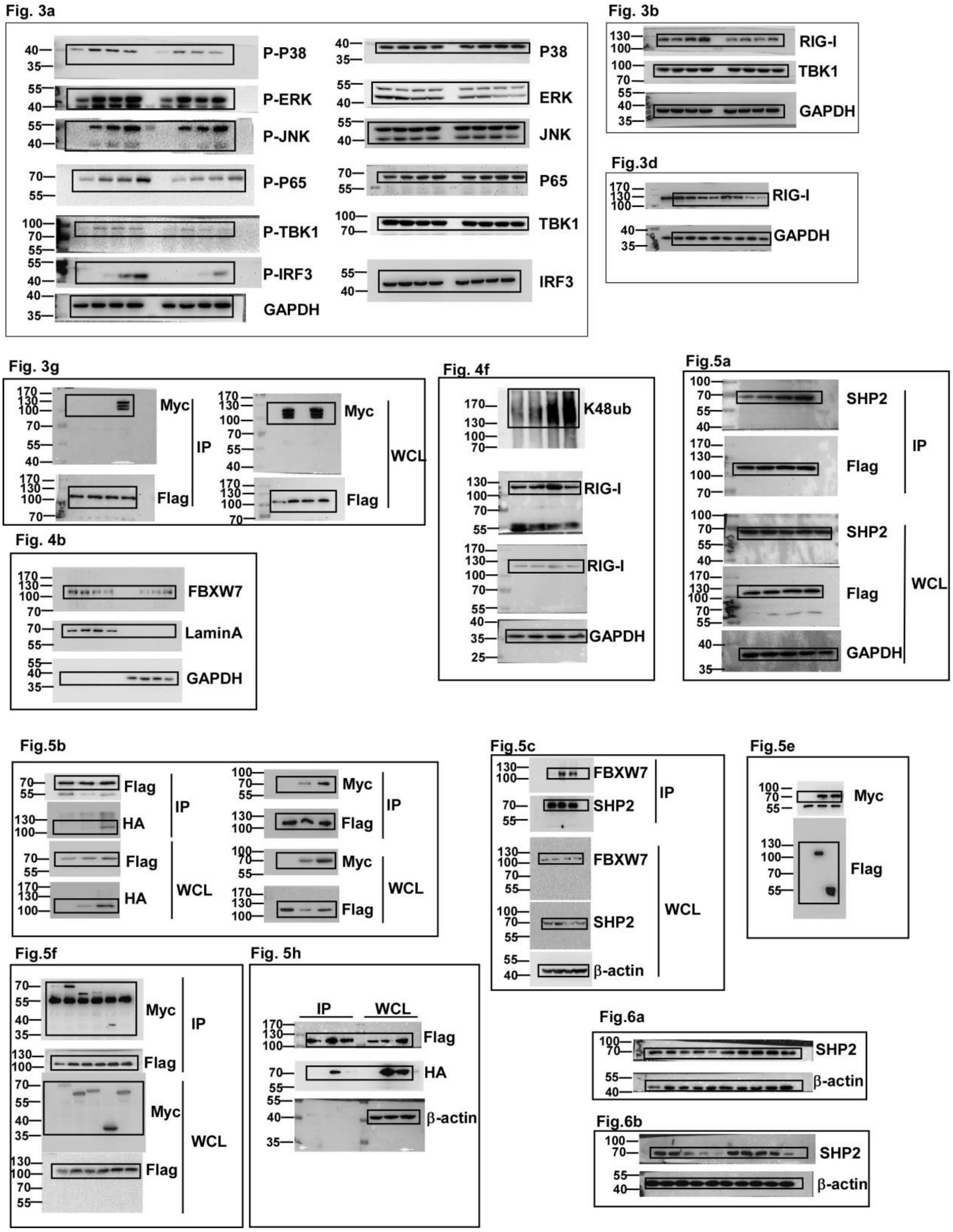

Supplementary Figure 9. Uncropped scans of the western blots related to Figure 3-5

Supplementary Figure 10

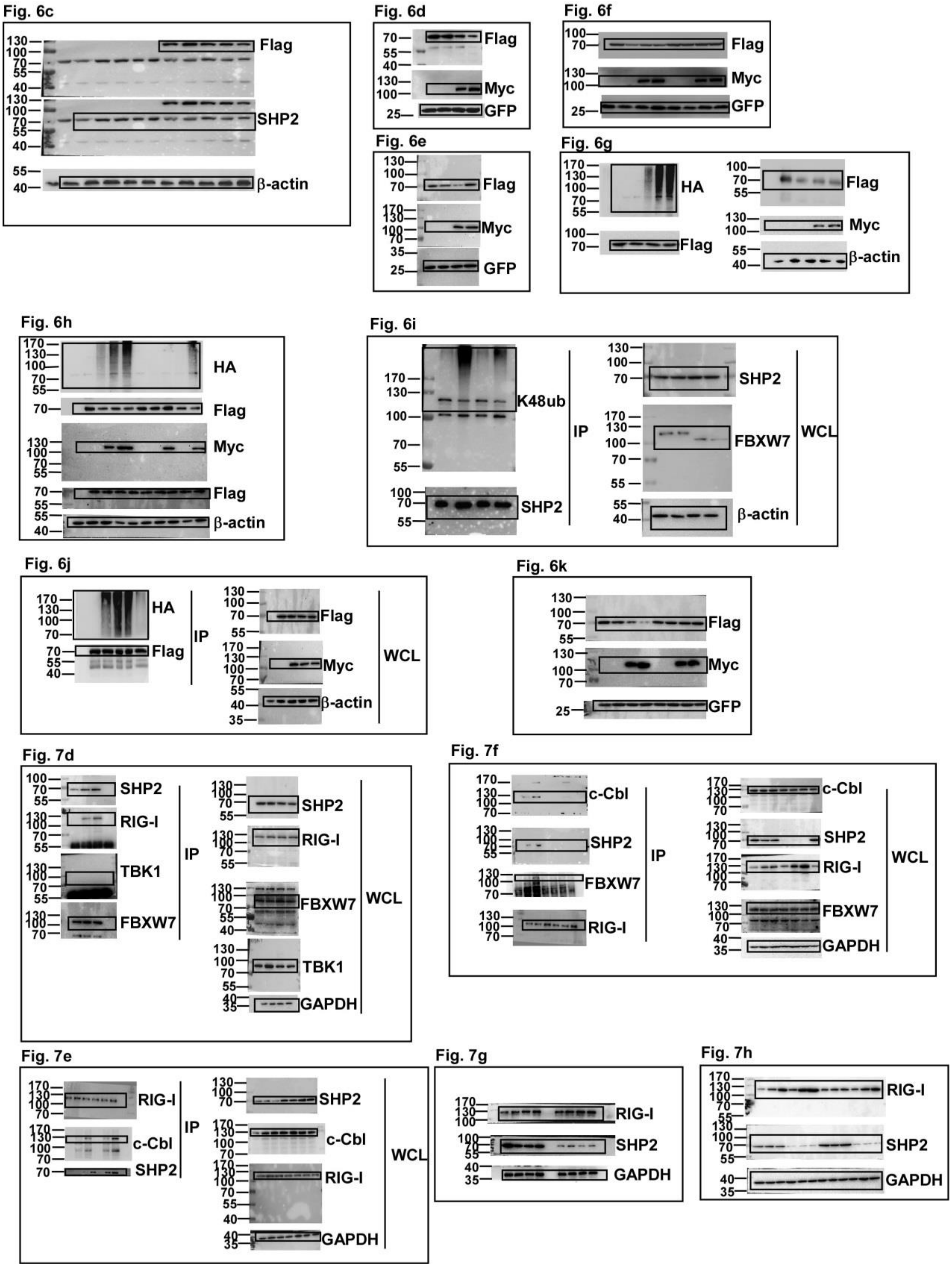

Supplementary Figure 10. Uncropped scans of the western blots related to Figure 6-7

Supplementary Figure 11

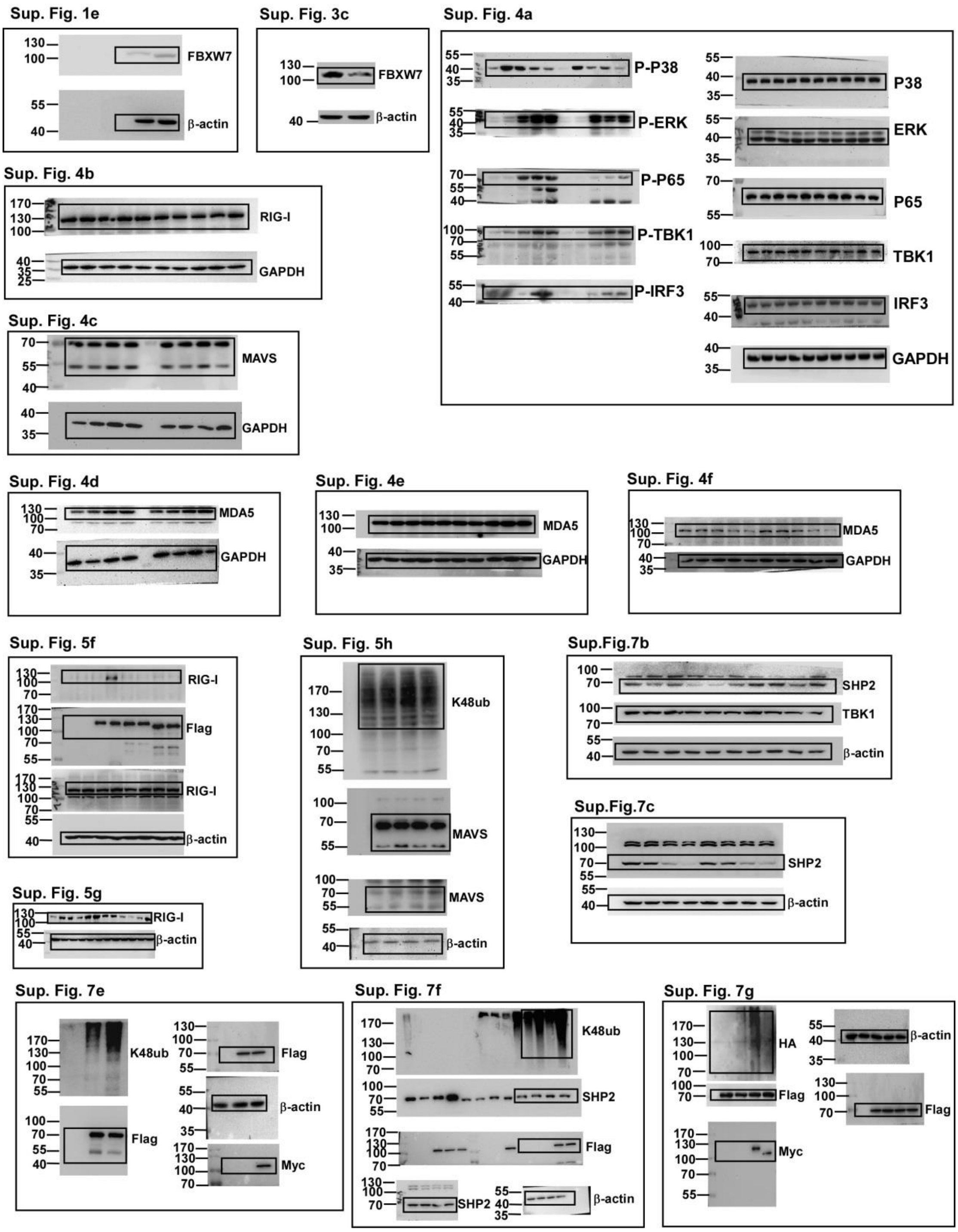

Supplementary Figure 11. Uncropped scans of the western blots related to Supplementary Figure 1-7

Supplementary Figure 12

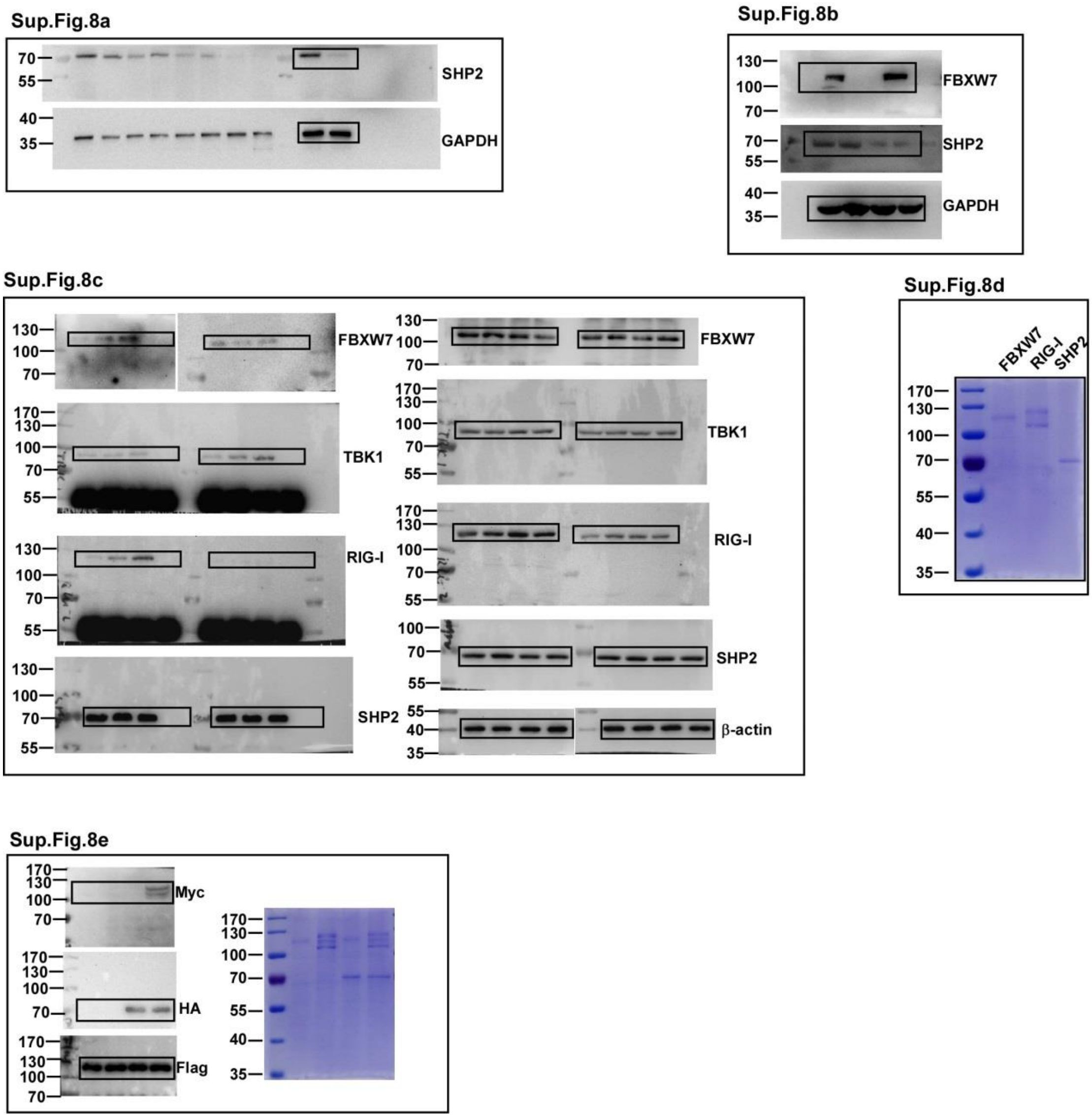

Supplementary Figure 12. Uncropped scans of the western blots related to Supplementary Figure 8

Supplementary Table 1. PCR Primers for expressing vectors, related to experimental procedures

mFBXW7 forward primer, 5'-AATTTGCGGCCGCaATGAATCAGGAACTG-3';
mFBXW7 reverse primer, 5'-GGCaagcttTTTCATGTCCACATCAAAGT-3';
mFBXW7-WD40 domain forward primer,
5'GGAATTCGCCACCATGCTCTGGAGAGAGAAATGT-3';
mFBXW7-WD40 domain forward primer,
5'-CCCAAGCTTCCTTTCATGTCCACATCAAAGTC-3';
mSHP2 forward primer, 5'-CCGGAATTCATGACATCGCGGAGATGGTTTCAC -3';
mSHP2 reverse primer, 5'-CGGGGTACCCCTCTGAAACTCCTCTGCTGCTGCAT -3';
mSHP2 K91R forward primer, 5'-GCTGAAAGAGCGGAATGGAGATGTTATCGA-3';
mSHP2 K91R reverse primer, 5'- TCGATAACATCTCCATTCCGCTCTTTCAGC-3';
mSHP2 K178R forward primer, 5'-GCTGAAAGAGCGGAATGGAGATGTTATCGA-3';
mSHP2 K178R reverse primer, 5'- TCGATAACATCTCCATTCCGCTCTTTCAGC-3';
mSHP2 S554 558A forward primer,
5'-TGGACCAGACAGCGGGTGATCAGGCGCCCCTGCCA-3';
mSHP2 S554 558A reverse primer,
5'-TGGCAGGGGCGCCTGATCACCCGCTGTCTGGTCCA-3';
mSHP2 ΔNSH forward primer,
5'-ATGACATCGCGGTGTGCAGACCCTACCTCTGAA-3';
mSHP2 ΔNSH reverse primer,
5'-TTCAGAGGTAGGGTCTGCACACCGCGATGTCAT-3';
mSHP2 ΔCSH forward primer,
5'-AACGACGGCAAGTCCAAAGTACTCGTATCAATGCTG-3';
mSHP2 ΔCSH reverse primer,
5'-AATTTCAGCAGCATTGATACGAGTTTCAGAGGTAGG-3';
mSHP2 ΔPTP forward primer,
5'-TGAGACCACAGATAAAGTCAAGCAGTACATAGAGAC-3';
mSHP2 ΔPTP reverse primer,
5'-TCCGGCGCTGCAGCGTCTCTATGTACTGCTTGACTTTATC-3';
mSHP2 ΔC-terminal forward primer, 5'-
TACATAGAGACGCTGCAGCGGGATCCGAGCTCGGTA-3';
mSHP2 ΔC-terminal reverse primer,
5'-AGTTTTACCGAGCTCGGATCCGTGCTGGACAGCC-3'.

Supplementary Table 2. RNA quantification, related to experimental procedures

Mouse FBXW7 forward primer, 5'-TGCAAAGTCTCAGATTATACC-3';

Mouse FBXW7 reverse primer, 5'-ACTTCTCTGGTCCGCTCCAGC-3';

Mouse SHP2 forward primer, 5'-AGGTCGGACAAGGAAACACA-3';

Mouse SHP2 reverse primer, 5'- CCCTCATTTCTGCACAGGGT-3';

Mouse IFN-β forward primer, 5'-ATGAGTGGTGGTTGCAGGC-3';

Mouse IFN-β reverse primer, 5'-TGACCTTTCAAATGCAGTAGATTCA-3';

Mouse IFN-α4 forward primer, 5'-TACTCAGCAGACCTTGAACCT-3';

Mouse IFN-α4 reverse primer, 5'-CAGTCTTGGCAGCAAGTTGAC-3';

Mouse IL-6 forward primer, 5'-TAGTCCTTCCTACCCCAATTTCC-3';

Mouse IL-6 reverse primer, 5'-TTGGTCCTTAGCCACTCCTTC-3';

Mouse RIG-I forward primer: 5'-CAGATCCGAGACACTAAAGGGA-3';

Mouse RIG-I reverse primer: 5'-TCCTCATCAGCCTTGCTTTCA-3'

VSV forward primer, 5'-ACGGCGTACTTCCAGATGG-3';

VSV reverse primer, 5'-CTCGGTTCAAGATCCAGGT-3';

Mouse GAPDH forward primer, 5'-AACTTTGGCATTGTGGAAGG-3';

Mouse GAPDH reverse primer, 5'-ACACATTGGGGGTAGGAACA-3';

Human FBXW7 forward primer, 5'-GACGCCGAATTACATCTGTC-3';

Human FBXW7 reverse primer, 5'-GTAGCAGGTCTTTGGGTTC-3';

Human IFN-β forward primer, 5'- CATTACCTGAAGGCCAAGGA-3';

Human IFN-β reverse primer, 5'- CAATTGTCCAGTCCCAGAGG-3';

Human GAPDH forward primer, 5'- TCAAGAAGGTGGTGAAGCAG-3';

Human GAPDH reverse primer, 5'- GAGGGGAGATTCAGTGTGGT-3'
