# Supplementary figures and images for: Author Correction: E3 ligase FBXW7 is critical for RIG-I stabilization during antiviral responses
Source: Nat Commun. 2026 Mar 30;17:2961. doi: 10.1038/s41467-026-70926-x (PMC13035808; doi:10.1038/s41467-026-70926-x)

Corrected Fig. 1d-1e

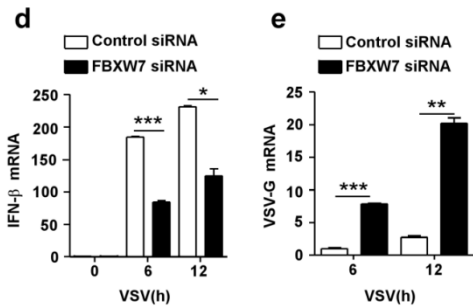

Corrected Supplementary Figure 1f-1g

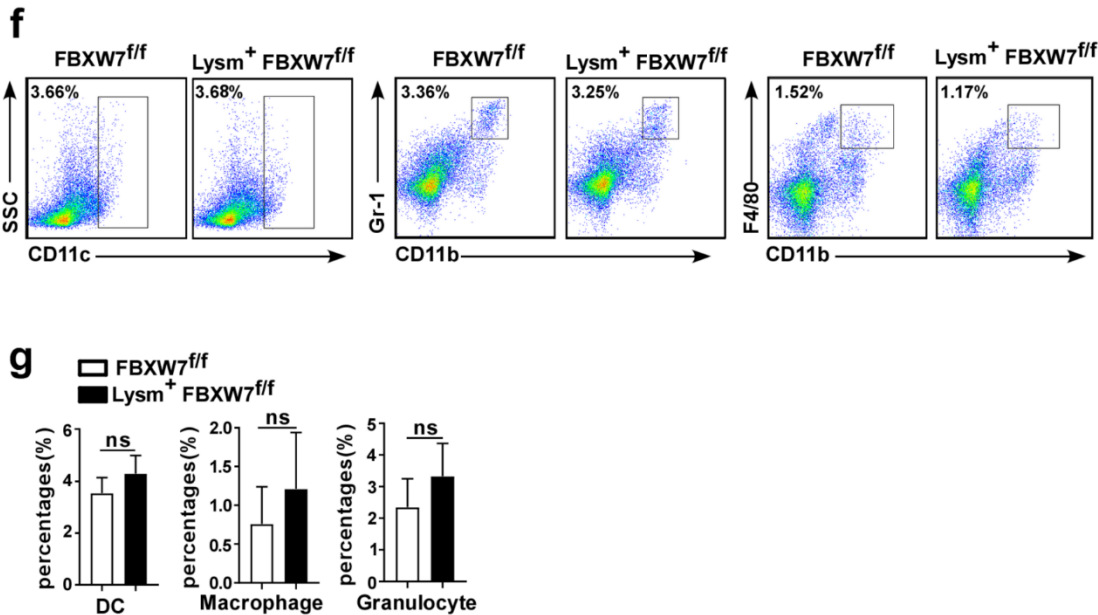

Corrected Supplementary Figure 6b

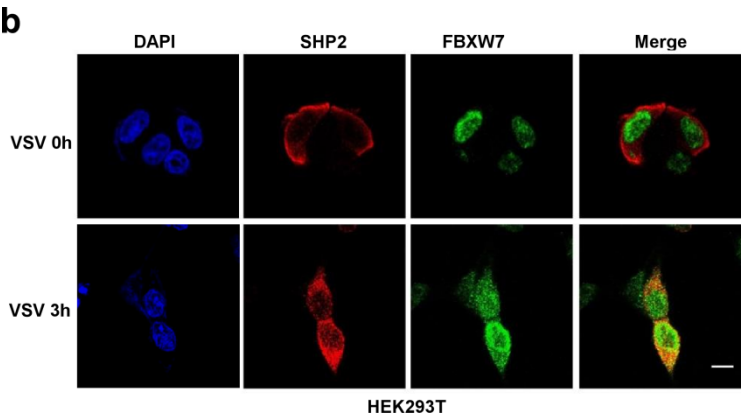

Supplement: Supplementary file 1 — Corrected Fig. 1d, e, Supplementary Figs. 1f, g, 6b [file 41467_2026_70926_MOESM1_ESM.pdf]
